# Case Report: The “atoll sign”: a case series on an unusual radiological pattern of immune-mediated pneumonitis

**DOI:** 10.3389/fimmu.2026.1791312

**Published:** 2026-06-16

**Authors:** Samuele Compagno, Alessandro Di Federico, Andrea De Giglio, Filippo Natali, Tommaso Abbate, Federica Ciccarese, Francesca Giunchi, Mattia Riefolo, Giorgia Dalpiaz, Stefania Damiani, Francesca Sperandi, Piero Candoli, Andrea Ardizzoni, Francesco Gelsomino

**Affiliations:** 1 Department of Medical and Surgical Sciences, University of Bologna, Bologna, Italy; 2 Medical Oncology, Istituto di Ricovero e Cura a Carattere Scientifico (IRCCS) Azienda Ospedaliero-Universitaria di Bologna, Bologna, Italy; 3 Istituto di Ricovero e Cura a Carattere Scientifico (IRCCS) Azienda Ospedaliero-Universitaria di Bologna, Bologna, Italy; 4 Department of Radiology, Istituto di Ricovero e Cura a Carattere Scientifico (IRCCS) Azienda Ospedaliero-Universitaria di Bologna, Bologna, Italy; 5 Pathology Unit, Istituto di Ricovero e Cura a Carattere Scientifico (IRCCS) Azienda Ospedaliero-Universitaria di Bologna, Bologna, Italy; 6 Department of Radiology, Bellaria Hospital, Bologna, Italy

**Keywords:** atoll sign, case-series, immune checkpoint inhibitor, immune-mediated pneumonitis, non-small cell lung cancer, organizing pneumonia

## Abstract

**Introduction:**

Immune checkpoint inhibitors (ICIs) have transformed the therapeutic landscape of advanced non-small cell lung cancer (NSCLC), offering durable survival in selected patients. However, ICIs can induce immune-related adverse events (irAEs), among which pneumonitis represents one of the most clinically significant due to its potential severity and diagnostic challenges. Different radiological patterns have been reported, although they may overlap with infectious or radiation-induced lung injury, complicating a timely diagnosis.

**Results:**

We report two cases of young patients with metastatic NSCLC and high PD-L1 expression who achieved long-term benefit from first-line pembrolizumab but were burdened by severe immune-related pneumonitis. Imaging findings in both patients were suggestive of organizing pneumonia associated with the presence of the so-called “atoll sign” (or reversed halo sign), a radiological feature rarely reported in the context of ICI-related pneumonitis. In both cases, the diagnosis was supported by imaging, microbiological tests, and histology. The subsequent administration of high-dose steroids led to rapid relief of symptoms associated with radiological improvement. Despite pembrolizumab discontinuation, both patients maintained durable systemic disease control.

**Conclusion:**

These cases emphasize the importance of recognizing uncommon radiological patterns such as the atoll sign in patients receiving ICIs, highlighting the need for vigilance even after prolonged therapy and underscoring the potential for sustained oncologic benefit despite treatment discontinuation.

## 1 Introduction

Non-small cell lung cancer (NSCLC) remains the leading cause of cancer-related death worldwide (1). The advent of immune checkpoint inhibitors (ICIs) targeting programmed death 1 (PD-1) and programmed death-ligand 1 (PD-L1) has markedly improved clinical outcomes in advanced disease, particularly for patients with high PD-L1 expression and no actionable oncogenic drivers, for whom pembrolizumab monotherapy is one of the current options as first-line treatment (2).

Despite their clinical benefits, ICIs are associated with a distinct spectrum of irAEs as a result of immune system hyperactivation. irAEs may affect any organ or system, but those detectable by CT are mainly pulmonary and abdominal, with pneumonitis being the most frequent (3). Several distinct radiographic patterns of pneumonitis have been observed, including nonspecific interstitial pneumonia, organizing pneumonia (OP), hypersensitivity pneumonitis, sarcoid-like reaction, acute interstitial pneumonia (AIP/ARDS), and transient asymptomatic pulmonary opacities (TAPOs); additionally, radiation recall phenomena may occur in previously irradiated fields. Among them, OP represents the most common pattern (3, 4).

OP is a clinico-radiological entity reflecting a reparative process of the lung parenchyma, which may be idiopathic or secondary to various insults, including infections, connective tissue disorders, or drugs. The high-resolution computed tomography (HRCT) pattern could widely vary, with patchy and often migratory consolidations (with subpleural, peribronchial distribution, or bandlike pattern), ground glass, and nodular opacities. When present, curvilinear perilobular opacities and the reversed halo (or atoll) sign are highly suggestive and could be helpful for diagnosis. The reversed halo sign is defined as a rounded ground-glass opacity surrounded by a partial or complete peripheral ring of consolidation (5, 6), which has only occasionally been reported in the context of ICI-induced pneumonitis (7, 8). Recognizing such atypical radiological presentations is critical for a timely diagnosis and management of immune-related pneumonitis.

Usually, in case of symptomatic or extensive immune-related pneumonitis, immunotherapy is withheld and corticosteroid started, followed by a slow tapering based on the patient’s response (9). In general, restarting immunotherapy is not recommended in severe cases.

In this study, we present two cases of young patients with metastatic NSCLC and high PD-L1 expression who achieved a long-lasting clinical benefit from first-line pembrolizumab but were burdened by severe immune-related pneumonitis, characterized by the onset of the reverse halo sign. These cases provide insight into the diagnostic and therapeutic challenges of this rare radiological pattern and contribute to the growing body of literature on immune-related lung injury.

Patients included expressed written consent to anonymized data collection and publication. The study was conducted in accordance with the Declaration of Helsinki.

## 2 Case description

### 2.1 Case 1

A 40-year-old woman, a former smoker (20 pack-years), was diagnosed in November 2019 with stage IVB NSCLC adenocarcinoma with pleomorphic features of the left lung. At baseline, metastatic sites included bilateral hilar and mediastinal lymph nodes, pleura, lungs, peritoneum, bone, and brain. Molecular profiling revealed a KRAS (p.G12A) mutation, and the PD-L1 tumor proportion score (TPS) was 90%. Eastern Cooperative Oncology Group (ECOG) performance status (PS) was 0 (**Table 1**).

**Table 1:** Summary of clinical course, immune-related pneumonitis features, and management in the two reported cases.

Characteristic	Case 1	Case 2
Age at diagnosis	40	45
Sex	Female	Male
History of tobacco use	Former smoker	Former smoker
Comorbidities	No comorbidities	Prior surgery for mixed carcinoma of the testis; appendicectomy; knee surgery for ligament repair.
Histology	NSCLC with pleomorphic features (CAM 5.2+, HMB45-, MART1-, TTF-1-)	Adenocarcinoma (TTF-1+, p40-)
PD-L1 TPS (Ventana – SP263)	90%	70%
NGS results Oncomine Focus Assay – Thermo-Fisher Scientific (Kit RUO)	KRAS (p.G12A)	Negative
1st line therapy	Pembrolizumab (carboplatin and pemetrexed chemotherapy was added to the ICI after documented primary progressive disease)	Pembrolizumab
Radiotherapy	Yes (single brain metastasis)	Yes (rib)
Response	Initial PD to pembrolizumab monotherapy. CR (after addition of chemotherapy)—oligo-PD - subsequent CR while on pembrolizumab monotherapy.	Complete metabolic response
ICI-related toxicities (grade) before pneumonitis	No	Cutaneous (G2)
Time from ICI initiation to pulmonary toxicity	65 months	10 months
Symptoms	Cough, dyspnea, and fever	Cough, dyspnea, and fever
Grade of pulmonary toxicity	G3	G2
BAL results [HSV-1, HSV-2, HHV-6, VZV, CMV, Mycobacterium tuberculosis complex (PCR), respiratory viruses (multiplex real-time PCR), galactomannan, actinomycetes, Nocardia, Pneumocystis jirovecii, aerobic bacteria, fungi, Aspergillus spp. DNA, and mycobacteria (smear and culture)]	Negative	Negative
BAL cellularity	Increased cellularity with 20% monocyte– macrophage population and 73% lymphocytes; CD4/CD8 inversion with 47% activated lymphocytes (CD3+ HLA-DR+).	Increased cellularity with predominance of the monocyte–macrophage population (34%) and 48% lymphocytes; CD4/CD8 inversion with 43% activated lymphocytes (CD3+ HLA-DR+), and 25% CD8+CD57b+ elements
Cellularity/histology	Lung parenchyma showing features of organizing pneumonia. PAS staining was negative.	Lung parenchyma with fibrotic organization, moderately thickened septa, and Masson bodies.
Corticosteroids treatment (prednisone 1 mg/kg/day)	Yes	Yes
Outcome		
Clinical	Symptom resolution	Symptom resolution
Radiological	Residual fibrotic bundle in the left base	Residual mild ground-glass
ICI resumption	No	No

BAL, bronchoalveolar lavage; CAM 5.2, cytokeratin antibody (clone CAM 5.2); CD, cluster of differentiation; CR, complete response; G, grade according to CTCAE 5.0; HLA-DR, human leukocyte antigen – DR isotype; HSV, herpes simplex virus; HHV-6, human herpesvirus 6; ICI, immune checkpoint inhibitor; NGS, next- generation sequencing; PAS, periodic acid–Schiff; PD, progressive disease; PD-L1, programmed death-ligand 1; PCR, polymerase chain reaction; TPS, tumor proportion score; TTF-1, thyroid transcription factor-1; VZV, varicella zoster virus.

**Figure 1:**
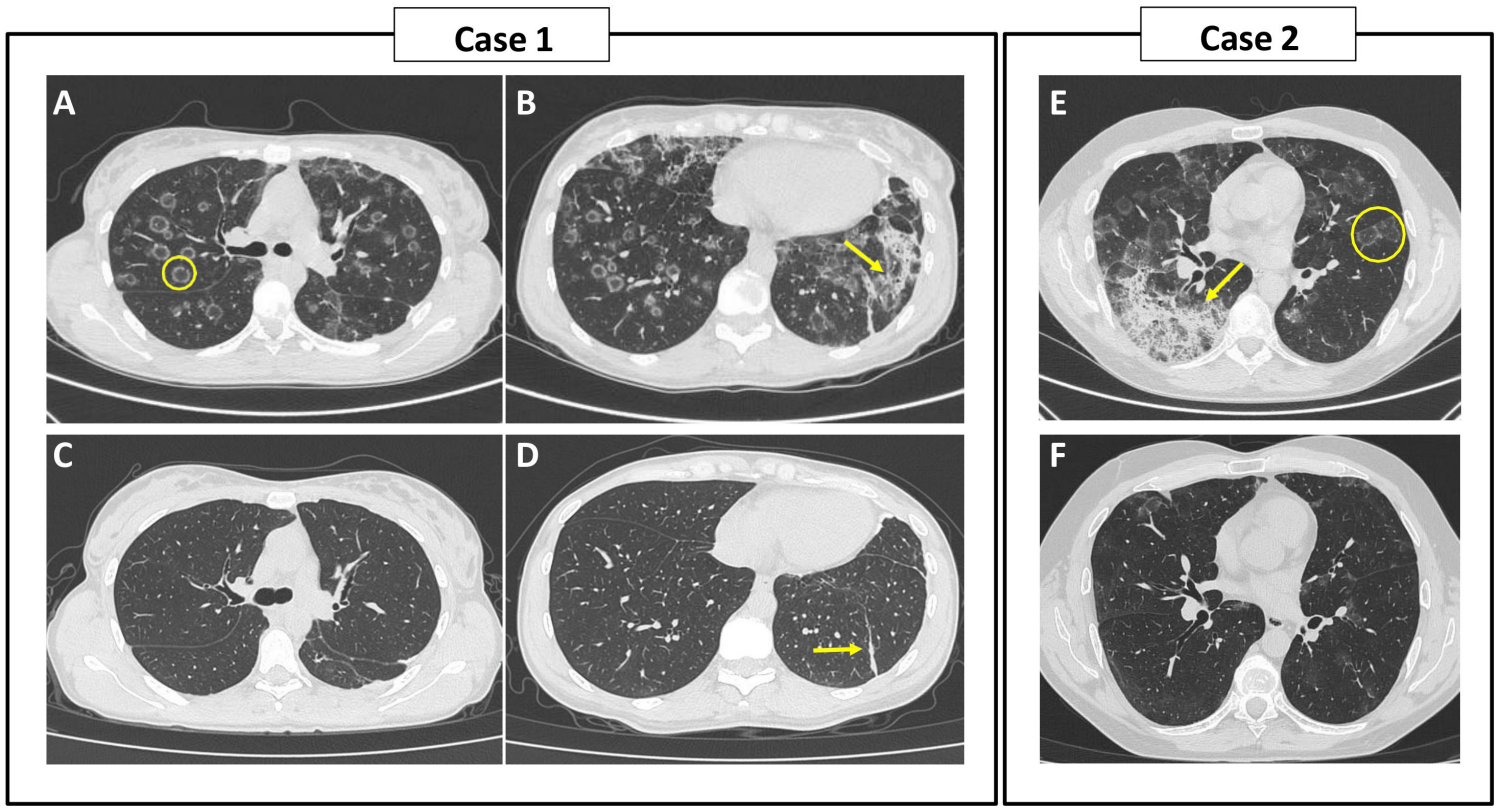
Case 1: multiple bilateral rounded ground-glass opacities with reversed halo sign [**(A)**-circle], associated with a bandlike subpleural consolidation at the left lower lobe [**(B)**-arrow]. Ground glass opacities showed random distribution (both central and peripheral), without cranio-caudal predominance (upper or lower lobes). No other extra-parenchymal ancillary findings (such as pleural effusion or lymphadenopathies) were detected. After corticosteroid therapy, complete regression of atoll sign **(C)**, with residual fibrotic bundle in the left lung base **(D)**. Case 2: wide consolidation at the apical segment of the right lower lobe [**(E)**-arrow], associated with multiple bilateral ground glass opacities with atoll sign [**(E)**-circle]. After therapy, complete regression, with residual mild ground-glass **(F)**. All CT scans were performed using a 128-slice CT scanner (Ingenuity; Philips) before and after contrast media injection, with the following technical parameters: tube voltage, 120 kV; tube current modulation, 100–250 mAs; spiral pitch factor, 1.224; collimation width, 64 × 0.625. For lung parenchyma evaluation, high-resolution reconstructions were obtained using a convolution kernel (Y-SHARP) at a slice thickness of 1 mm.

First-line pembrolizumab monotherapy was started, but the patient experienced rapid disease progression after one cycle. The addition of platinum-based chemotherapy to pembrolizumab, followed by maintenance pembrolizumab, led to a rapid clinical benefit, accompanied by documented durable systemic and intracranial response (10). In 2023, the patient developed a single brain metastasis that was treated with stereotactic radiotherapy, achieving a complete response (**Table 1**). Serial CT scans performed during pembrolizumab treatment consistently demonstrated sustained systemic disease control without evidence of thoracic progression. After more than five years of pembrolizumab therapy, a CT scan performed in February 2025 showed the appearance of subpleural ground-glass opacities (GGOs) in the context of otherwise stable disease. Although the patient was initially asymptomatic, two months later she developed grade 1 cough, low-grade fever, and grade 2 dyspnea. A follow-up CT scan showed progression of the bilateral GGOs with a reversed halo sign associated with a subpleural consolidation (**Figures 1A, B**). Pembrolizumab was discontinued.

**Figure 2:**
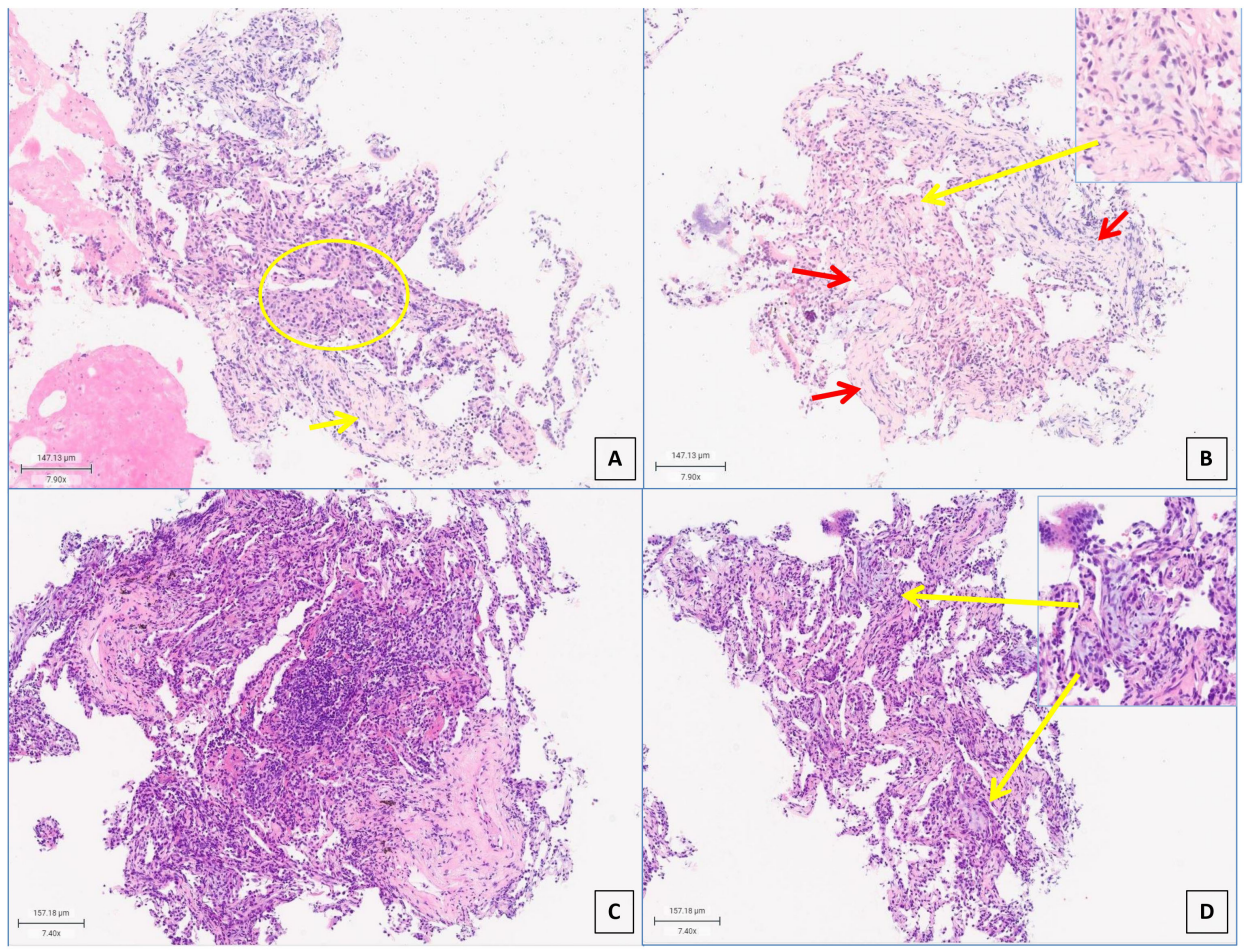
Case 1: **(A)** hematoxylin-eosin (H&E) lung biopsy with architectures preserved, with fibrosis of the septa (arrow) and macrophage accumulation in the alveoli (circle). **(B)** parenchymal fibrosis with fibroblastic proliferation (yellow arrow and close-up) and fibrosis (red arrow). Case 2: **(C)** H&E lung biopsy with rich chronic inflammation, fibrosis, fibrin. **(D)** dense aggregate of fibroblasts with immature collagen (arrow and close-up).

The patient subsequently underwent bronchoscopy with bronchoalveolar lavage (BAL) and transbronchial biopsy prior to the initiation of any treatment; microbiological analyses were negative. The transbronchial biopsies (TBBs) obtained small pieces of lung parenchyma and bronchial wall tissue; the lung architecture was preserved with fibrous septa and airspace organization with fibroblastic proliferation, resembling a Masson body. The alveolar space presented macrophage accumulation. Periodic acid–Schiff (PAS) stain was negative. These findings were suggestive of organizing pneumonia (**Table 1**; **Figures 2A, B**). No evidence of malignant cells was observed.

After the endoscopic procedure, corticosteroid therapy with oral prednisone at 1 mg/kg/day was initiated, along with empirical antibiotic treatment with amoxicillin/clavulanate, resulting in rapid clinical improvement and resolution of symptoms. Amoxicillin/clavulanate was stopped after six days, while prednisone was tapered throughout six weeks. By October 2025, a complete radiological resolution of pneumonitis was obtained (**Figures 1C, D**), while the patient maintained an ECOG PS of 0 and an ongoing systemic and intracranial disease remission supporting a non-neoplastic etiology of the pulmonary findings.

### 2.2 Case 2

A 45-year-old man, a former smoker (15 pack-years), presented in April 2024 with haemoptysis and thoracic pain. CT and 18F-FDG-PET-CT scans revealed a solid consolidation at the left lower lobe with pleural metastases and left rib involvement. The histology report was consistent with lung adenocarcinoma, with PD-L1 TPS 70%, and no driver oncogene alteration was found by genomic sequencing. The patient had an ECOG PS of 0, and the disease was staged as IVA.

First-line pembrolizumab was initiated in June 2024, alongside palliative radiotherapy to the rib lesion. By January 2025, an 18F-FDG-PET-CT demonstrated a complete metabolic response. In February 2025, the patient developed a fever and cough. HRCT showed consolidations within the prior radiation field, raising suspicion for radiation pneumonitis. BAL was negative for pathogens, and the patient achieved partial clinical improvement following corticosteroid therapy with oral prednisone at 0.5 mg/kg/day. After two months, pembrolizumab was resumed, but, during steroid tapering, the patient developed recurrent respiratory symptoms, consisting of cough and dyspnea, and fever.

In May 2025, HRCT demonstrated a new consolidation in the right lower lobe, outside the previously irradiated field, associated with multiple bilateral ground-glass opacities with an atoll sign (**Figure 1E**), suggesting a distinct process from the initial radiation pneumonitis. Bronchoscopy and cultures from BAL excluded infection. TBBs collected some small pieces of lung parenchyma with diffuse chronic inflammation (lymphocytes) and fibrosis. In the septa, enlarged for the fibrosis, there were some dense aggregates of fibroblasts with immature collagen (Masson bodies) that confirmed the diagnosis of OP (**Table 1** and **Figures 2C D**). Biopsy samples, obtained from the newly developed pulmonary opacities, did not reveal malignant infiltration.

Pembrolizumab was discontinued, and high-dose corticosteroid therapy was initiated with oral prednisone at 1 mg/kg/day, followed by a gradual tapering schedule over a total duration of three months. Empirical antibiotic therapy with amoxicillin/clavulanate was also administered for seven days. This approach resulted in rapid symptom resolution and progressive radiological improvement. By November 2025 CT scan showed near-complete resolution of pneumonitis (**Figure 1F**). Serial imaging confirmed persistent complete response without evidence of disease progression, supporting the diagnosis of organizing pneumonia rather than tumor progression. The patient remained in complete response with ECOG PS 0 under close surveillance.

## 3 Discussion

Immune-related pneumonitis is a clinically significant toxicity related to ICIs, with heterogeneous clinical and radiological manifestations that make diagnosis and management challenging. Both cases presented here highlight the occurrence of the atoll sign, a radiological finding rarely reported in the context of ICI-related pneumonitis (7, 8).

The atoll sign, first described in cryptogenic organizing pneumonia, consists of a central ground-glass opacity surrounded by partial or complete peripheral consolidation. Although non-specific, its recognition in the setting of immunotherapy should prompt consideration of ICI-related organizing pneumonia in the differential diagnosis (11). For both patients, diagnosis was supported by histological confirmation, excluding infection and disease progression.

Timing is another key aspect. While many irAEs occur early on in treatment, our two patients developed pneumonitis after prolonged exposure to pembrolizumab over five years in one case and ten months in the other case. This underscores the need for long-term surveillance, as pulmonary irAEs may emerge late, even after years of an apparently well-tolerated therapy.

Management in both cases followed international guidelines (12), which suggest ICI discontinuation and initiation of high-dose corticosteroids followed by a slow tapering. Both patients achieved a significant symptomatic and radiological improvement, reinforcing the effectiveness of a timely corticosteroid therapy.

Notably, despite permanent discontinuation of pembrolizumab, both patients maintained durable systemic control, with complete disease remission in one and persistent partial remission in the other. This suggests that long-lasting antitumor immunity may persist beyond ICI withdrawal, consistent with prior studies (13, 14).

Although the atoll sign represents a typical radiological feature of organizing pneumonia, its specific documentation in the context of ICI–related pneumonitis remains limited. Accumulating case reports may help better define its clinical significance. Recognizing this imaging presentation is important to differentiate immune-related lung toxicity from infection or radiation-induced injury, thereby facilitating appropriate management. A multidisciplinary approach remains essential for accurate diagnosis and optimal treatment of irAEs.

This report has several strengths, including histological confirmation of organizing pneumonia, exclusion of infectious etiologies, and radiological follow-up. However, the small number of cases and the descriptive nature of this report limit any inference regarding the incidence or causal relationship of this radiological presentation in ICI–related pneumonitis.

## 4 Patient perspective

Both patients reported a clear subjective improvement in respiratory symptoms following corticosteroid therapy, allowing them to resume normal daily activities. In Case 1, the patient described initial concern regarding the onset of respiratory symptoms after long-term immunotherapy but reported reassurance after diagnostic clarification. During corticosteroid treatment, she experienced sleep disturbances, which she identified as the most bothersome side effect. In Case 2, the patient expressed concern about the recurrence of respiratory symptoms after initial improvement. During corticosteroid therapy, he reported weight gain, which he perceived as impacting his quality of life and social relationships. Both patients stated that they were adequately informed about treatment decisions, including immunotherapy discontinuation, and reported overall satisfaction with clinical management. At the most recent follow-up (April 2026), both patients considered their condition stable and compatible with normal daily functioning.

## 5 Conclusion

These two cases describe ICI-related organizing pneumonia presenting with the atoll sign in patients with metastatic NSCLC treated with pembrolizumab. Organizing pneumonia is a recognized manifestation of immune-related pneumonitis, and the reversed halo sign is a typical radiological feature of this histopathologic pattern. However, its occurrence in the specific context of ICI-related pneumonitis appears to be infrequently documented.

Early recognition and prompt corticosteroid initiation enabled favorable pulmonary outcomes while durable oncologic benefit persisted despite immunotherapy discontinuation. Further documentation of such cases is needed to improve diagnostic accuracy, guide clinical decision-making, and refine management strategies for rare ICI-related pulmonary toxicities.

## Data Availability

The original contributions presented in the study are included in the article/supplementary material. Further inquiries can be directed to the corresponding author.
